# Characterization of Retail Conventional, Organic, and Grass Full-Fat Butters by Their Fat Contents, Free Fatty Acid Contents, and Triglyceride and Fatty Acid Profiling

**DOI:** 10.3390/foods6040026

**Published:** 2017-03-31

**Authors:** Annemieke M. Pustjens, Rita Boerrigter-Eenling, Alex H. Koot, Maikel Rozijn, Saskia M. van Ruth

**Affiliations:** 1 RIKILT Wageningen Research, Wageningen University and Research, P.O. Box 230, 6700 AE Wageningen, The Netherlands; Rita.boerrigter-eenling@wur.nl (R.B.-E.); Alex.koot@wur.nl (A.H.K.); Maikel.rozijn@wur.nl (M.R.); Saskia.vanruth@wur.nl (S.M.v.R.); 2 Food Quality and Design, Wageningen University and Research, P.O. Box 17, 6700 AA Wageningen, The Netherlands

**Keywords:** authenticity, butter, fat content, free fatty acids, fatty acid composition, grass, organic, triglyceride profile

## Abstract

In the Netherlands, butter is produced from milk originating from three different production systems: conventional, organic, and grass-fed cows. The aim of the current study was to characterize these types of butters, and pinpoint distinct compositional differences. Retail conventional (*n* = 28), organic (*n* = 14), and grass (*n* = 12) full-fat butters were collected during the winter and summer seasons. Samples were analyzed for their fat content, free fatty acid (FFA) content, and triglyceride (TG) and fatty acid (FA) profiles. The fat content was significantly lower in conventional butters than in organic butters and the FFA content was significantly lower in conventional butters compared with grass butters. Also, organic butters differed significantly from their conventional counterparts with regard to their TG and FA profiles. The TG profiles of the organic and grass butters did not differ significantly. The FA profiles of grass butters were less distinct, since only a few FAs differed significantly from conventional (six FAs) and organic (eight FAs) butters.

## 1. Introduction

In the Netherlands, butter is produced from milk originating from three different production systems: conventional production, organic production, and a specialized part of the conventional system in which cows are grass-fed. According to European regulations (EC Regulation 889/2008 [1]), organic milk comes from cows that can roam freely (weather and health permitting) and are fed organic feed which contains at least 60% roughage. Their diet cannot contain genetically modified ingredients. Furthermore, the use of synthetic fertilizers, synthetic medicines, antibiotics for preventive use, and synthetic growth hormones are not allowed in organic farming. Organic farming is more expensive than conventional farming, for example because organic cows have lower milk yields. Therefore, other initiatives which present a positive image for consumers have appeared on the market, such as for dairy products made from milk produced by grazing cows. For example, grass butter is made from milk produced by cows with access to fresh grass. In the past, grass butter was only produced during a short period of the year when cows are transferred from the stables to the pasture, but nowadays all the milk produced by cows that are fed fresh grass is used. However, in Europe, there are no legal requirements for the use of the term “grass butter”.

Organic butter and grass butter are assumed to be more animal and environmentally friendly and sometimes perceived as being healthier by the consumer. The applicability of several methods to authenticate the types of milk fat, in general, has been evaluated: analytical methods that were found to be capable of distinguishing conventional and organic milk fat were, for example, the contents of α-tocopherol and β-carotene [2,3]; the stable isotope ratio of carbon and the content of α-linolenic acid (C18:3*n*3) [4,5]; ^1^H-NMR and ^13^C-NMR [6]; and triglyceride (TG) and fatty acid (FA) profiles [7,8,9,10]. The fatty acid profiles of retail organic milk were found to be significantly different from the FA profiles of conventional and pasture milk [8]. Published data on butter showed that conventional and organic butter had a significantly different FA profile [11]. Data on the composition of grass butter are missing. Seasonal effects on the fat composition of butter and milk have been investigated before for Irish [12] and Polish [13] conventional butters and for Dutch raw milk [14]. Significant differences in the TG and FA profile between butters produced in summer and in winter were found, which may result from the alternative feeding systems employed in summer, i.e., pasture versus indoor feeding [13].

Apart from different feeding regimes for the cows, processing can also specifically alter the fat composition. For example, microbial lipases can be added or they can be produced by the microorganisms used for the inoculation of the cream, as is done in Europe and Asia. These lipases can catalyze the interesterification of FAs to obtain a desirable positional distribution of FAs on the TGs [15]. In addition, milk fat can be fractionated into portions of TGs. This can either be done by crystallization of melted fat, or methods based on the different solubility and volatility of groups of TGs [16]. These fractions can be added again to the final product. These alterations can be performed in order to obtain a final product with the desired sensory (mainly taste and color), physical (e.g., spreadability versus firmness), chemical, and nutritional characteristics.

The aim of the current study was to characterize retail full-fat conventional, organic, and grass butters, and pinpoint distinct differences in their fat composition. Fat content, free fatty acid content, as well as triglyceride and fatty acid profiles were examined.

## 2. Materials and Methods

### 2.1. Samples

In total, 54 samples of salted and unsalted full-fat butters of conventional, organic, and grass-fed production origin were purchased from eight different supermarkets in the Netherlands. Fourteen conventional butters from 11 different brands were collected, of which four were salted and 10 unsalted. Seven organic butters from five different brands were collected, of which two were salted and five were unsalted. Six grass butters from five different brands were collected, of which one was salted and five were unsalted. This set of samples was purchased on two different occasions: once in November 2015 and once in June 2016. As soon as the butters were purchased, they were transferred into screw-top plastic bottles and stored in the refrigerator (4 °C) prior to analyses.

### 2.2. Chemicals

All chemicals used were of analytical grade purchased from Merck (Darmstadt, Germany), Sigma-Aldrich (St. Louis, MO, USA), or VWR International (Radnor, PA, USA), unless stated otherwise.

### 2.3. Analytical Methods

#### 2.3.1. Fat Content

The fat content was determined gravimetrically in duplicate according to NEN-EN-ISO 17189:2003 | IDF 194 [17]. In short, fat was extracted from the sample (4–6 g) using petroleum ether. The solvent and fat phase were separated and the solvent was removed by evaporation. The mass of substances extracted was determined.

#### 2.3.2. Free Fatty Acid Content

The content of free fatty acids (FFA) was determined titrimetrically in duplicate, conforming with NEN-ISO 1740:2004 | IDF 6 [18]. In short, a sample (containing 5–10 g fat) was solubilized in petroleum ether 60–80%. Sodium thymol blue solubilized in 2-propanol was added as an indicator. Titration took place in the presence of nitrogen gas using tetra-*n*-butyl ammonium hydroxide until the color changed.

#### 2.3.3. Triglyceride Analysis

Triglyceride analysis was carried out in duplicate according to ISO 17678 | IDF 202 [19] by gas chromatography with a flame ionization detector (GC-FID), as described by Capuano et al. [7]. TGs were identified by comparing their retention times with those found in standard mixtures. Results were expressed as normalized peak areas (% total TG + cholesterol).

#### 2.3.4. Fatty Acid Analysis

Fatty acid analysis was carried out according to ISO 16958 [20] by capillary gas chromatography after direct transesterification of the samples. Fatty acids were identified by comparing their retention times with those of standard mixtures (GLC 36 mix (Nu-Check Prep INC, Elysian, MN, USA)). Results were expressed as normalized peak areas (% on total fatty acids).

### 2.4. Data Analysis

The normality of the distributions of fat contents, FFA contents, TG profiles, and FA profiles within the groups—conventional, organic, and grass butters—were checked using the Shapiro–Wilk test. Distributions appeared to be non-normal (Shapiro–Wilk: *p* < 0.05). Therefore, the Kruskal–Wallis test for group comparison was performed among the groups. If the Kruskal–Wallis test was significant (*p* < 0.05), pairwise comparisons between groups were subsequently performed by means of the Mann–Whitney U test. SPSS version 23.0.0.2 (IBM Corp., Armonk, NY, USA) was used to perform those tests. The same procedure was followed to check significant differences between samples purchased in winter and samples purchased in summer and between salted and unsalted butter samples.

Principal Component Analysis (PCA) was performed using The Unscrambler (Version X 10.3, CAMO Software, Oslo, Norway). The (*I* × *J*) data matrix consisted of *I* = 54 rows (butter samples) and *J* = 17 columns for the TGs and *J* = 35 columns for the FAs. Raw data were auto-scaled prior to PCA.

## 3. Results

In the present study, retail conventional, organic, and grass full-fat butters were analyzed for their fat contents, FFA contents, TG profiles, and FA profiles. Samples were collected in winter and in summer in order to identify robust differences in the production systems. Where substantial differences between the butters purchased in winter and those purchased in summer were found, these are described in the text. No significant differences between unsalted and salted butters were observed, therefore, no distinction is made between these groups in the results section.

### 3.1. Fat Content and Free Fatty Acids

In Table 1, the fat contents and FFA contents of conventional, organic, and grass full-fat butters are shown. Unsalted butter should contain at least 82% (m/m) fat, whereas salted butter should contain at least 80% (m/m) according to EC Regulation 1898/2005 [21]. All samples analyzed in this study met this requirement. The fat contents were significantly lower in conventional butters compared to organic butters. The fat content of grass butters was not significantly different from conventional and organic butters, and winter and summer butters also showed similar fat contents.

The FFA content was significantly higher in grass butters than in conventional butters. Fresh grass contains plant lipase, which could cause the increase in FFA, as has been described for milk from grass fed cows [22]. The FFA content in organic butters was not significantly different from both conventional and grass butters. The FFA content tended to be higher in summer rather than in the winter period; this is probably because conventional and organic cows are also grass-fed in summer.

### 3.2. Triglyceride Profiles

**Figure 1:**
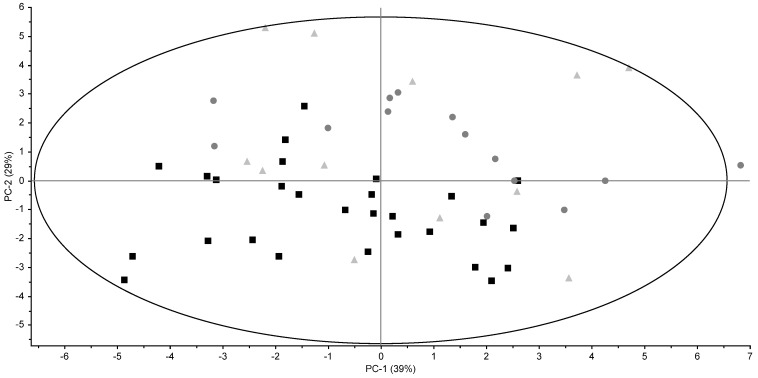
Principal Component Analysis (PCA) score plots of TG profiles for conventional (black square), organic (dark grey round), and grass (light grey triangle) retail full-fat butters.

The average TG and cholesterol profiles of conventional, organic, and grass full-fat butters are shown in Table 2. The most abundant TGs in butter were those containing 36, 38, and 50 acyl carbon atoms (CN36, CN38, and CN50, respectively). These were also found to be the most abundant TGs in Polish butters [13]. Differences between types of butter were small, however, some TGs were significantly different in the different types of butter. The TGs CN40, CN44, CN46, and CN48 were found to be significantly different in conventional butters compared with organic and grass butters. CN24, CN26, and CN54 were significantly different in conventional butters compared with organic butters, but not with grass butters. The results of the TG profiles are also visualized in a Principal Component Analysis (PCA) plot (Figure 1). Conventional and organic butters tend to be distinguishable, but grass butters are not. The distinction of pasture milk from conventional milk has also been proven difficult based on the TG profiles [8]. Generally, no obvious seasonal effects on the TG profiles were observed.

### 3.3. Fatty Acid Profiles

The samples were also subjected to fatty acid profiling, the results of which are shown in Table 3. The most abundant FA in butter was palmitic acid (C16:0), followed by myristic (C14:0), stearic (C18:0), and oleic acid (C18:1*n*9), as was also described in American [11], Irish [12], and Polish [13] butters. The FA C16:0 was significantly higher in conventional butters than in organic and grass butters. Furthermore, 18 other fatty acids showed significant differences between conventional and organic butters. Between conventional and grass butters, only three FAs were significantly different. Alpha-linolenic acid (C18:3*n*3) was found to be the only FA that is statistically different in all three types of butter. Molkentin [4,23] showed that organic dairy had a relative α-linolenic acid greater than 0.50%. This is also the case for the samples analyzed in this study, in which organic butters had an average α-linolenic acid content of 0.73%, while conventional butters had an average α-linolenic acid content of 0.43%. The α-linolenic acid content of grass butters was also just above the threshold with 0.54% on average. The contents of conjugated linolenic acid (C18:2 CLA), which is known for its anticarcinogenic effect, and trans C18:1 were significantly lower in conventional butters than in organic and grass butters, as has been reported previously for Italian butters and other dairy products [2]. No significant differences between organic and grass butters were found for these FAs.

**Figure 2:**
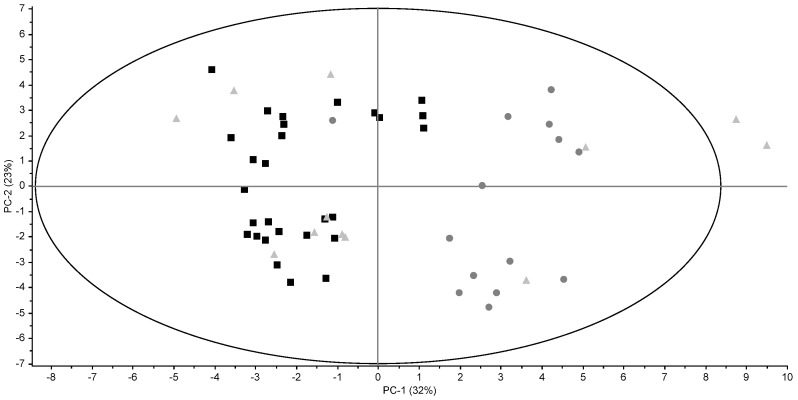
PCA score plots of FA profiles for conventional (black square), organic (dark grey round), and grass (light grey triangle) retail full-fat butters.

Results of the FA profile are also visualized in a PCA plot (Figure 2). Conventional and organic butters were well distinguished, but grass butters were not. Previously published results on dairy produce from grass-fed cows were not consistent; on the one hand, fresh grass feeding has been shown to significantly affect the FA composition of the milk produced [9,24]. On the other hand, differences in the FA composition between conventional and pasture retail milk were found to be negligible [8]. Differences between organic butters on the one hand and conventional and grass butters on the other hand were more pronounced in winter than in summer. This is probably due to the fact that in summer conventional and organic cows are also grass-fed.

**Figure 3:**
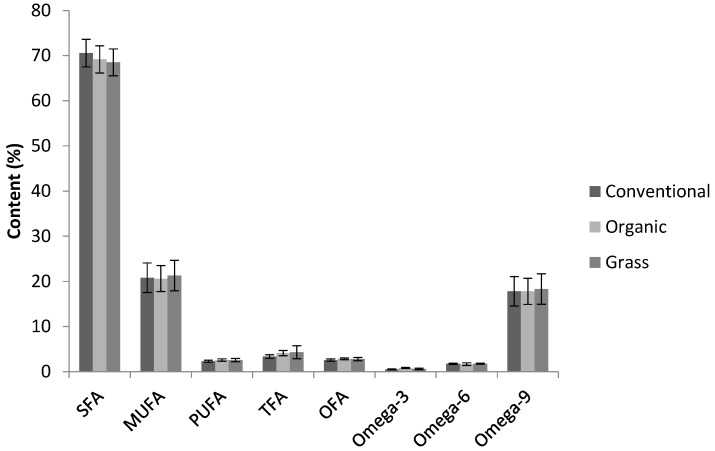
Comparing the contents of saturated fatty acids (SFA), monounsaturated fatty acids (MUFA), polyunsaturated fatty acids (PUFA), *trans* fatty acids (TFA), and other fatty acids (OFA) in conventional, organic, and grass butters. Error bars represent standard deviations.

For groups of FAs, the results are shown in Figure 3. Polyunsaturated fatty acids (PUFA), *trans* fatty acids (TFA), and omega-3 FAs were significantly higher in organic butters than in conventional butters. For PUFA and omega-3 FAs, this has been described before for conventional and organic milk [25]. The results for grass butters were, again, less obvious which is likely due to its relatively flexible production specifications.

SFA and PUFA contents as well as omega-3 and omega-6 FA contents tended to be higher in butters purchased in summer than those purchased in winter for all types of butter; MUFA and omega-9 FAs showed the opposite trend. Delgadillo-Puga also described a significant difference between samples collected in January and June in SFA and MUFA in conventional raw milk and for PUFA in organic raw milk [25]. An increase in PUFA and omega-3 in grass-fed dairy was expected, since these groups of FAs are more abundant in grass than in conventional feed [26].

## 4. Conclusions

In conclusion, the present study showed that the triglyceride (TG) and fatty acid (FA) profiles of organic butters differed significantly from their conventional counterparts. The distinction of grass butters was not possible based on TG and FA profiles. To continue the verification of the production type of butter based on fat content and composition, more samples should be collected to provide insight into differences between years, seasons, and brands. To be able to evaluate the seasonal effect in greater detail, butters could be collected in consultation with producers directly after production.

## Figures and Tables

**Table 1:** Average content ± standard deviation of fat and free fatty acids of conventional (C), organic (O), and grass (G) retail full-fat butters: mean concentration, standard deviation and significant differences between butter types (Kruskal–Wallis).

	C (*n* = 28)	O (*n* = 14)	G (*n* = 12)	*p*-Value
Fat content (m/m %)	82.33 ± 0.56 ^a^	83.16 ± 0.68 ^b^	82.74 ± 0.48 ^ab^	0.001
Free fatty acids (% oleic acid)	0.148 ± 0.017 ^a^	0.164 ± 0.036 ^ab^	0.166 ± 0.020 ^b^	0.035

^a, b^ Distributions of fat content and free fatty acid content in groups C, O, and G with different superscript letters within a row are significantly different (Mann–Whitney U test, *p* < 0.05).

**Table 2:** Average triglyceride (TG) composition of conventional (C), organic (O), and grass (G) retail full-fat butters: mean concentration, standard deviation and significant differences between butter types (Kruskal–Wallis).

TG	C (*n* = 28)	O (*n* = 13)	G (*n* = 12)	*p*-Value
CN24	0.06 ± 0.02 ^a^	0.09 ± 0.03 ^b^	0.06 ± 0.02 ^a^	0.007
Cholesterol	0.29 ± 0.05 ^a^	0.32 ± 0.02 ^a^	0.31 ± 0.04 ^a^	0.155
CN26	0.33 ± 0.02 ^a^	0.34 ± 0.02 ^a^	0.33 ± 0.03 ^a^	0.144
CN28	0.73 ± 0.04 ^a^	0.78 ± 0.04 ^b^	0.77 ± 0.07 ^ab^	0.006
CN30	1.39 ± 0.08 ^a^	1.43 ± 0.10 ^a^	1.45 ± 0.12 ^a^	0.240
CN32	2.86 ± 0.12 ^a^	2.86 ± 0.15 ^a^	2.93 ± 0.19 ^a^	0.543
CN34	6.30 ± 0.19 ^a^	6.13 ± 0.25 ^a^	6.25 ± 0.28 ^a^	0.132
CN36	11.11 ± 0.25 ^a^	10.91 ± 0.29 ^a^	10.88 ± 0.47 ^a^	0.225
CN38	12.23 ± 0.17 ^a^	12.27 ± 0.14 ^a^	12.36 ± 0.27 ^a^	0.285
CN40	9.58 ± 0.13 ^a^	9.91 ± 0.17 ^b^	9.92 ± 0.39 ^b^	<0.001
CN42	7.08 ± 0.19 ^a^	7.11 ± 0.17 ^a^	7.05 ± 0.17 ^a^	0.777
CN44	6.91 ± 0.21 ^a^	6.68 ± 0.24 ^b^	6.69 ± 0.14 ^b^	0.002
CN46	7.68 ± 0.28 ^a^	7.34 ± 0.23 ^b^	7.38 ± 0.27 ^b^	0.001
CN48	9.19 ± 0.18 ^a^	8.79 ± 0.20 ^b^	8.84 ± 0.39 ^b^	<0.001
CN50	11.01 ± 0.26 ^a^	10.93 ± 0.31 ^a^	10.77 ± 0.34 ^a^	0.089
CN52	9.18 ± 0.52 ^a^	9.44 ± 0.56 ^a^	9.40 ± 0.50 ^a^	0.361
CN54	4.08 ± 0.45 ^a^	4.67 ± 0.56 ^b^	4.60 ± 0.84 ^ab^	0.007

^a, b^ Distributions of each individual TG in groups C, O, and G with different superscript letters within a row are significantly different (Mann–Whitney U test, *p* < 0.05).

**Table 3:** Average fatty acid composition of conventional (C), organic (O), and grass (G) retail full-fat butters; mean concentration, standard deviation and significant differences between butter types (Kruskal–Wallis).

Fatty Acid	C (*n* = 28)	O (*n* = 14)	G (*n* = 12)	*p*-Value
C4:0	3.99 ± 0.61 ^a^	4.02 ± 0.53 ^a^	3.99 ± 0.49 ^a^	0.970
C6:0	2.33 ± 0.13 ^a^	2.39 ± 0.11 ^a^	2.35 ± 0.11 ^a^	0.255
C8:0	1.36 ± 0.05 ^a^	1.40 ± 0.04 ^b^	1.40 ± 0.06 ^b^	0.031
C10:0	3.13 ± 0.18 ^a^	3.19 ± 0.20 ^a^	3.25 ± 0.27 ^a^	0.407
C12:0	4.05 ± 0.24 ^a^	3.78 ± 0.23 ^b^	4.03 ± 0.30 ^a^	0.008
C14:0	12.00 ± 0.54 ^a^	11.82 ± 0.36 ^a^	11.75 ± 0.45 ^a^	0.377
C14:1*n*5	1.08 ± 0.07 ^a^	1.03 ± 0.07 ^a^	1.07 ± 0.13 ^a^	0.274
C15:0	1.16 ± 0.06 ^a^	1.25 ± 0.05 ^b^	1.19 ± 0.10 ^ab^	0.001
C15:1*n*5	0.00 ± 0.00 ^a^	0.00 ± 0.00 ^a^	0.00 ± 0.00 ^a^	1.000
C16:0	31.77 ± 1.29 ^a^	30.19 ± 1.94 ^b^	29.66 ± 2.55 ^b^	0.002
C16:1*n*7	1.72 ± 0.08 ^a^	1.56 ± 0.08 ^b^	1.68 ± 0.23 ^ab^	0.001
C17:0	0.50 ± 0.05 ^a^	0.55 ± 0.04 ^b^	0.51 ± 0.06 ^ab^	0.009
C17:1*n*8	0.21 ± 0.03 ^a^	0.23 ± 0.03 ^ab^	0.25 ± 0.03 ^b^	0.006
C18:0	10.11 ± 0.53 ^a^	10.33 ± 0.65 ^a^	10.24 ± 0.73 ^a^	0.550
trans C18:1	2.89 ± 0.35 ^a^	3.58 ± 0.54 ^b^	3.72 ± 1.22 ^b^	<0.001
C18:1	17.75 ± 3.27 ^a^	17.72 ± 2.88 ^a^	18.24 ± 3.36 ^a^	0.550
trans C18:2	0.45 ± 0.06 ^a^	0.52 ± 0.09 ^b^	0.57 ± 0.22 ^b^	0.008
C18:2*n*6	1.49 ± 0.14 ^a^	1.45 ± 0.27 ^a^	1.51 ± 0.15 ^a^	0.508
C18:2 CLA	0.54 ± 0.13 ^a^	0.86 ± 0.19 ^b^	0.78 ± 0.36 ^b^	<0.001
C18:3*n*6	0.03 ± 0.05 ^a^	0.01 ± 0.03 ^a^	0.05 ± 0.06 ^a^	0.069
trans C18:3	0.00 ± 0.00 ^a^	0.00 ± 0.00 ^a^	0.00 ± 0.00 ^a^	0.021
C18:3*n*3	0.43 ± 0.09 ^a^	0.73 ± 0.11 ^b^	0.54 ± 0.15 ^c^	<0.001
C20:0	0.10 ± 0.06 ^a^	0.13 ± 0.03 ^b^	0.07 ± 0.06 ^a^	0.001
C20:1*n*9	0.04 ± 0.01 ^a^	0.04 ± 0.01 ^a^	0.04 ± 0.01 ^a^	0.536
C20:2*n*6	0.01 ± 0.00 ^a^	0.02 ± 0.00 ^a^	0.01 ± 0.01 ^a^	0.232
C20:3*n*6	0.06 ± 0.01 ^a^	0.05 ± 0.01 ^b^	0.05 ± 0.01 ^ab^	<0.001
C20:3*n*3	0.00 ± 0.00 ^a^	0.00 ± 0.00 ^a^	0.00 ± 0.00 ^a^	0.003
C20:4*n*6	0.11 ± 0.01 ^a^	0.09 ± 0.01 ^b^	0.10 ± 0.01 ^a^	0.028
C20:5*n*3	0.04 ± 0.01 ^a^	0.06 ± 0.01 ^b^	0.05 ± 0.02 ^a^	<0.001
C22:0	0.05 ± 0.01 ^a^	0.06 ± 0.01 ^b^	0.05 ± 0.01 ^a^	0.003
C22:1*n*9	0.00 ± 0.00 ^a^	0.00 ± 0.00 ^a^	0.00 ± 0.00 ^a^	0.511
C22:2*n*6	0.03 ± 0.01 ^a^	0.04 ± 0.00 ^b^	0.04 ± 0.02 ^ab^	<0.001
C22:6	0.00 ± 0.00 ^a^	0.00 ± 0.00 ^a^	0.00 ± 0.00 ^a^	1.000
C24:0	0.03 ± 0.01 ^a^	0.04 ± 0.00 ^b^	0.02 ± 0.01 ^a^	0.001
C24:1*n*9	0.00 ± 0.00 ^a^	0.00 ± 0.00 ^a^	0.00 ± 0.00 ^a^	0.928

^a, b, c^ Distributions of each individual fatty acid in groups C, O, and G with different superscript letters within a row are significantly different (Mann–Whitney U test, *p* < 0.05).
